# *Lycium Barbarum* Polysaccharides Reduce Neuronal Damage, Blood-Retinal Barrier Disruption and Oxidative Stress in Retinal Ischemia/Reperfusion Injury

**DOI:** 10.1371/journal.pone.0016380

**Published:** 2011-01-26

**Authors:** Suk-Yee Li, Di Yang, Chung-Man Yeung, Wing-Yan Yu, Raymond Chuen-Chung Chang, Kwok-Fai So, David Wong, Amy C. Y. Lo

**Affiliations:** 1 Eye Institute, Li Ka Shing Faculty of Medicine, The University of Hong Kong, Hong Kong; 2 Department of Anatomy, Li Ka Shing Faculty of Medicine, The University of Hong Kong, Hong Kong; 3 Research Center of Heart, Brain, Hormone and Healthy Aging, Li Ka Shing Faculty of Medicine, The University of Hong Kong, Hong Kong; 4 St. Paul's Eye Unit, The Royal Liverpool University Hospital, Liverpool, United Kingdom; Chinese University of Hong Kong, Hong Kong

## Abstract

Neuronal cell death, glial cell activation, retinal swelling and oxidative injury are complications in retinal ischemia/reperfusion (I/R) injuries. *Lycium barbarum* polysaccharides (LBP), extracts from the wolfberries, are good for “eye health” according to Chinese medicine. The aim of our present study is to explore the use of LBP in retinal I/R injury. Retinal I/R injury was induced by surgical occlusion of the internal carotid artery. Prior to induction of ischemia, mice were treated orally with either vehicle (PBS) or LBP (1 mg/kg) once a day for 1 week. Paraffin-embedded retinal sections were prepared. Viable cells were counted; apoptosis was assessed using TUNEL assay. Expression levels of glial fibrillary acidic protein (GFAP), aquaporin-4 (AQP4), poly(ADP-ribose) (PAR) and nitrotyrosine (NT) were investigated by immunohistochemistry. The integrity of blood-retinal barrier (BRB) was examined by IgG extravasations. Apoptosis and decreased viable cell count were found in the ganglion cell layer (GCL) and the inner nuclear layer (INL) of the vehicle-treated I/R retina. Additionally, increased retinal thickness, GFAP activation, AQP4 up-regulation, IgG extravasations and PAR expression levels were observed in the vehicle-treated I/R retina. Many of these changes were diminished or abolished in the LBP-treated I/R retina. Pre-treatment with LBP for 1 week effectively protected the retina from neuronal death, apoptosis, glial cell activation, aquaporin water channel up-regulation, disruption of BRB and oxidative stress. The present study suggests that LBP may have a neuroprotective role to play in ocular diseases for which I/R is a feature.

## Introduction

Many ocular diseases such as acute glaucoma and amaurosis fugax may be associated with ischemia/reperfusion (I/R). During I/R, there are breakdown of blood-retinal barrier (BRB), accumulation of fluid within retina, oxidative stress and neuronal death [1], [2]. Under normal conditions, Muller cells play an important role in maintaining water and ion homeostasis via a variety of water and ion transport channels [1], [3], [4]. Aquaporin-4 (AQP4) is one of such water transport channels expressed in Muller cells and astrocytes [5], [6]. It has been shown that Muller cells and AQP4 are involved in retinal swelling which is associated with neuronal death [1], [2].

*Lycium barbarum* (Gouqizi, *Fructus Lycii*, Wolfberry), a traditional Chinese medicine, has been used for centuries in the East to maintain “eye health and nourish the liver and kidney”, and to balance “Yin” and Yang” in the body [7]. *Lycium barbarum* has a high content of polysaccharides which is approximately 40% by dry mass; therefore studies have been focused to the liquid fraction of the berries, the *Lycium barbarum* polysaccharides (LBP) [7]. LBP is derived from an extraction process that involved the removal of the lipid soluble components such as zeaxanthin and other carotenoids with alcohol [8]. Previous studies have shown that LBP can modulate the immune function [9], [10], act against the effects of aging and oxidation [11], [12], protect against liver damage [13], lower blood glucose level [14], and reduce the side effects of chemotherapy and radiotherapy [15], [16]. LBP also exerts beneficial effects in animal models of ocular diseases. Previously, LBP has been shown to protect retinal ganglion cells (RGCs) in an animal model of chronic ocular hypertension [17]. However, the protective effects of LBP in minimizing neuronal death, glial cell activation, BRB disruption and oxidative stress after I/R injury have not been investigated. Our present study aimed to determine whether LBP could limit the damages from retinal I/R injury in mice.

## Materials and Methods

### Ethics Statement

The use of animals in this study was conducted according to the requirements of the Cap. 340 Animals (Control of Experiments) Ordinance and Regulations, and all relevant legislation and Codes of Practice in Hong Kong. All the experimental and animal handling procedures were approved by the Faculty Committee on the Use of Live Animals in Teaching and Research in The University of Hong Kong (CULATR #1870-09).

### Animals

C57BL/6N male mice (10–12 weeks old) were used in the present study. The animals were kept in a temperature-controlled room with 12-hr light/12-hr dark cycle in the Laboratory Animal Unit of The University of Hong Kong.

### Pre-treatment with LBP

LBP extracts were prepared as described previously [8]. The extracts were freeze-dried as powder for storage. For experimental use, LBP solution was freshly prepared by dissolving the powder in phosphate-buffered saline (PBS; 0.01 M; pH 7.4). Animals were divided into two groups (n = 7/group) and were treated with either PBS as the vehicle or LBP (1 mg/kg). All animals were fed orally by gavage with either PBS or LBP once daily for 1 week prior to the induction of retinal ischemia.

### Animal model of retinal I/R

Animals were anesthetized (2% halothane in 70% N_2_O/30% O_2_ for induction and 1% halothane in 70% N_2_O/30% O_2_ for maintenance) and the rectal temperature were kept at 37±0.5°C. The internal carotid artery (ICA) is one of the bifurcations of the common carotid artery and provides blood supply to the cerebral regions. It also provides blood supply to the eye as the ophthalmic artery is a branch of the pterygopalatine artery (PPA) which originates from the ICA [18]–[21]. Unilateral retinal ischemia was induced by inserting an 8/0 nylon monofilament coated with vinyl polysiloxane impression material (3 M Dental Products, St. Paul, MN) through the right external carotid artery (ECA) into the right ICA. Both the right common carotid artery and right ECA were ligated to avoid anastomoses between the ophthalmic artery and the external carotid artery. Retinal ischemia was maintained for 2 hrs after which the filament was pulled out to allow reperfusion for 22 hrs [22].

### Tissue processing

Animals were sacrificed by an overdose of sodium pentobarbital and tissue processing was performed. Eyeballs were immediately enucleated, fixed, dehydrated and embedded in paraffin. Seven-µm cross-sections were cut using a microtome (Microm HM 315R, Heidelberg, Germany).

### Histological evaluation

Procedures described in our recently published study were adopted [22]. Retinal sections of the vehicle-treated eyes and the LBP-treated eyes were deparaffinized and stained with hematoxylin and eosin (H&E). Four pictures, two from central (100 µm from the optic nerve head) and two from peripheral retina (100 µm from the peripheral end of retina) of each retinal section were captured. The field size of each picture was approximately 0.07 mm^2^. Viable cells in ganglion cell layer (GCL) of the central and peripheral retina were counted. Cells with pyknotic nuclei were regarded as dead cells and were not counted in the counting. Retinal swelling in our I/R model has been shown to be due to the increase in inner retinal thickness [23]; therefore, retinal swelling was assessed by measuring the inner retinal thickness from the inner limiting membrane (ILM) to the inner nucleus layer (INL). For consistency, only retinal sections with optic nerve stumps were used [22].

### Terminal deoxynucleotidyl transferase biotin-dUTP nick end labeling (TUNEL)

Apoptosis was examined by TUNEL assay (DeadEnd Fluorometric TUNEL system, Promega, Madison, WI) as previously described [22]. To verify the TUNEL staining was localized in the nucleus, sections were counterstained with 4,6-diamidino-2-phenylindole (DAPI) after the reaction. Four pictures (two from the central and two from the peripheral retina) were captured from each retinal section. The field size of each picture was approximately 0.07 mm^2^. TUNEL-positive nuclei in both GCL and inner nuclear layer (INL) were counted.

### Immunohistochemistry (IHC)

Antigen retrieval was performed by incubation with proteinase K following deparaffinization of sections. Non-specific binding sites were blocked with normal goat serum for 1 hr at room temperature and then incubated with primary antibodies listed in Table 1 overnight at 4°C. Procedures of signal detection were adopted from our previous studies [22], [23]. Semi-quantitative analysis was carried out to assess the immunoreactivity [24], [25]. Slides were randomly coded and examined in a double-blinded approach. IHC scores were given according to the intensity as well as the number of cells and synaptic terminals stained. Score 1 represented the weakest immunoreactivity while score 5 indicated the highest immunoreactivity. Photomicrographs were captured under a light microscope (Eclipse 80i; Nikon, Tokyo, Japan) equipped with a digital camera (Diagnostic Instruments, Inc., Sterling Heights, MI).

**Table 1:** Primary antibodies.

Primary antibody name	Dilution	Catalogue #	Source
**Aquaporin-4**	1∶500	AB3594	Millipore
**Calretinin**	1∶1000	sc-11644	Santa Cruz Biotech.
**Glial fibrillary acidic protein (GFAP)**	1∶500	Z0334	Dako
**Neuronal nitric oxide synthase (nNOS)**	1∶1000	07-571	Upstate Biotech.
**Nitrotyrosine (NT)**	1200	06-284	Upstate Biotech.
**Poly(ADP-ribose) (PAR)**	1∶200	ALX-804-220	Alexis
**Protein kinase C-α (PKC-α)**	1∶1000	sc-208	Santa Cruz Biotech.

### IgG extravasations

Sections were blocked with Mouse on Mouse (MOM) blocking solution (MOM kit, Vector Laboratories, Burlingame, CA) and incubated with mouse biotinylated anti-mouse IgG secondary antibody (MOM kit) as previous described [26]. Signal was visualized by Vectastain ABC kit (Vector Laboratories) with 3.3′-diaminobenzidine tetrahydrochloride (Zymed, South San Francisco, CA). Leaky vessels were defined as vessels with immuno-positive IgG signals seen outside the endothelial vessel lining. The number of leaky and non-leaky blood vessels in the GCL and the outer plexiform layer (OPL) were counted.

### Study design and statistical analysis

The right eye of each animal was subjected to I/R, and the left eye was used as a non-ischemic control. The retinae of right eyes from animals pre-treated with vehicle were compared with those from the non-ischemic control left eyes of both groups. The retinae of right eyes from animals pre-treated with LBP were compared with those from animals pre-treated with vehicle. A double-blinded approach was used in all the experimental procedures, including cell counting and data analyses. Data were presented as mean ± SEM. One-way Analysis-of-variance (ANOVA) followed by the Bonferroni multiple comparison tests (Prism v4.0, GraphPad Software, Inc., San Diego, CA) was used in cell counting and IgG extravasations quantification. Kruskal-Wallis Test followed by the Dunn's multiple comparison tests (Prism v4.0) was performed in the semi-quantitative analyses of IHC scores. Statistically significant difference was set at p<0.05.

## Results

### Ganglion cell layer

**Figure 1:**
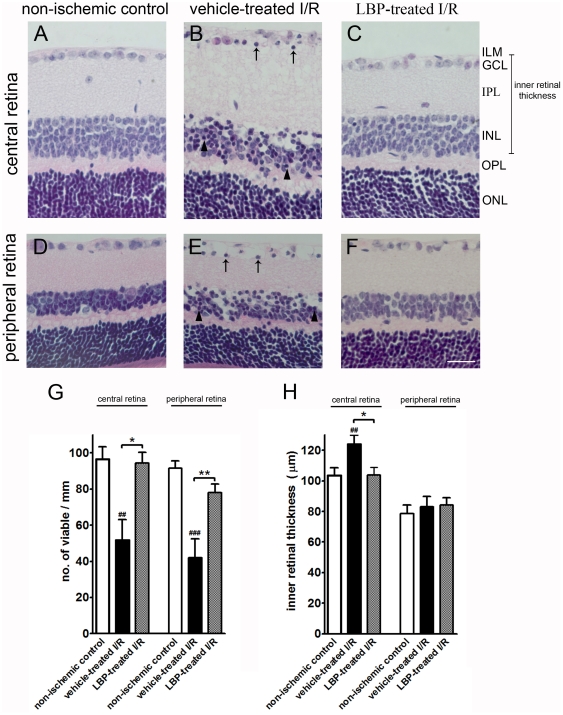
Higher cell count and increased retinal thickness in the LBP-treated I/R retina. (A–F) Representative photomicrographs of H&E stained retinal sections. (A–C) Central retina. (D–F) Peripheral retina. Pyknotic cells were observed in GCL (arrows in B & E) and INL (arrow heads in B & E) of the vehicle-treated I/R retina. Fewer pyknotic cells were observed in the LBP-treated I/R retina (C & F). (G) Viable cell count in GCL. Fewer viable cells were found in the vehicle-treated I/R retina when compared with the non-ischemic control retina (^##^p<0.01 in central retina; ###p<0.001 in peripheral retina). More viable cells were found in LBP-treated I/R retina when compared with the vehicle-treated I/R retina (*p<0.05 in central retina; **p<0.01 in peripheral retina). (H) Inner retinal thickness (from ILM to INL). There was an increase of retinal thickness in the central retina of vehicle-treated I/R mice when compared with that in the non-ischemic control mice (^##^p<0.01). However, there was a decrease of retinal thickness in the central retina of LBP-treated I/R mice when compared with that in the vehicle-treated I/R mice (*p<0.05). No change in inner retinal thickness of the peripheral retina was observed among groups (p>0.05). Scale bar, 25 µm. I/R, ischemia/reperfusion; LBP, Lycium barbarum polysaccharides; ILM, inner limiting membrane; GCL, ganglion cell layer; IPL, inner plexiform layer; INL, inner nuclear layer; OPL, outer plexiform layer; ONL, outer nuclear layer.

2-hr ischemia followed by 22-hr reperfusion caused severe tissue damage in the vehicle-treated retina. Many cells in the GCL and INL were pyknotic in the central and peripheral retina (Fig. 1B & 1E). The number of viable cells in GCL of the central and the peripheral retina was significantly lower in the vehicle-treated I/R mice compared with that in the non-ischemic control mice (central retina: 51.6±11.3 vs. 96.6±6.8 cells/mm, p<0.01; peripheral retina: 42.1±10.3 vs. 91.6±4.0 cells/mm, p<0.001) (Fig. 1G). However, pre-treatment of LBP diminished the damage of I/R (Fig. 1C & 1F). The number of viable cells in GCL in the central and the peripheral retina was higher in the LBP-treated I/R mcie compared with that in the vehicle-treated I/R mice (central retina: 94.4±5.9 vs. 51.6±11.3 cells/mm, p<0.05; peripheral retina: 78.1±4.6 vs. 42.1±10.3 cells/mm, p<0.01) (Fig. 1G).

### Retinal thickness

There was an increase in inner retinal thickness of the central retina in the vehicle-treated mice as compared with that in the non-ischemic control mice (central retina: 124.0±5.9 vs. 103.4±5 µm, p<0.01; peripheral retina: 83.1±6.7 vs. 78.7±5.4 µm, p>0.05) (Fig. 1H). On the other hand, there was a decrease in inner retinal thickness of the central retina in the LBP-treated I/R mice as compared with the vehicle-treated I/R mice (central retina: 103.8±5.0 vs. 124.0±5.9 µm, p<0.05; peripheral retina: 84.2±4.7 vs. 83.1±6.7 µm, p>0.05) (Fig. 1H).

### Apoptosis in GCL & INL

**Figure 2:**
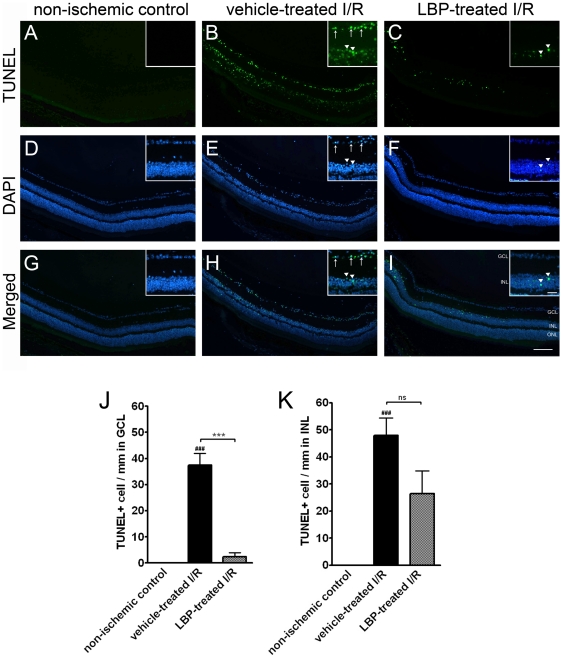
Fewer apoptosis in LBP-treated I/R retina. (A–C) TUNEL. (D–F) DAPI. (G–I) Merged images of TUNEL and DAPI. Insets are magnified views of the GCL and INL. (J, K) Quantification of TUENL-positive cells in GCL (J) and INL (K). In the vehicle-treated I/R retina (B), many TUNEL-positive cells were observed in both GCL (J, arrows in B) and INL (K, arrow heads in B) when compared with the non-ischemic control retina (A) (^###^p<0.001). In the LBP-treated I/R retina (C), fewer TUENL-positive cells were observed in GCL (J, ***p<0.001) when compared with the vehicle-treated I/R retina (B). Apparently less apoptosis was noted in INL in LBP-treated I/R retina (arrow heads in C) when compared with the vehicle-treated retina (B) although the difference is not statistically significant (K, p>0.05). Scale bars, 100 µm; Inset scale bars, 25 µm. ns, no significance.

Ischemia leads to apoptosis in the retina. More TUNEL-positive cells were observed in the GCL of the vehicle-treated I/R retina compared with that of the non-ischemic control retina (37.5±4.5 vs. 0 cells/mm, p<0.001) (Fig. 2B & 2J) and similarly in the INL (47.9±6.5 vs. 0 cells/mm, p<0.001) (Fig. 2B & 2K). Fewer apoptotic cells were found in GCL of the LBP-treated I/R retina when compared with that of the vehicle-treated I/R retina (2.4±1.4 vs. 37.5±4.5 cells/mm, p<0.001) (Fig. 2C & 2J). Slightly fewer TUNEL-positive cells were found in the INL of the LBP-treated I/R retina when compared with that of the vehicle-treated I/R retina; the difference however was not statistically significant (26.5±8.4 vs. 47.9±6.5 cells/mm, p>0.05) (Fig. 2C & 2K).

### Bipolar cells

**Figure 3:**
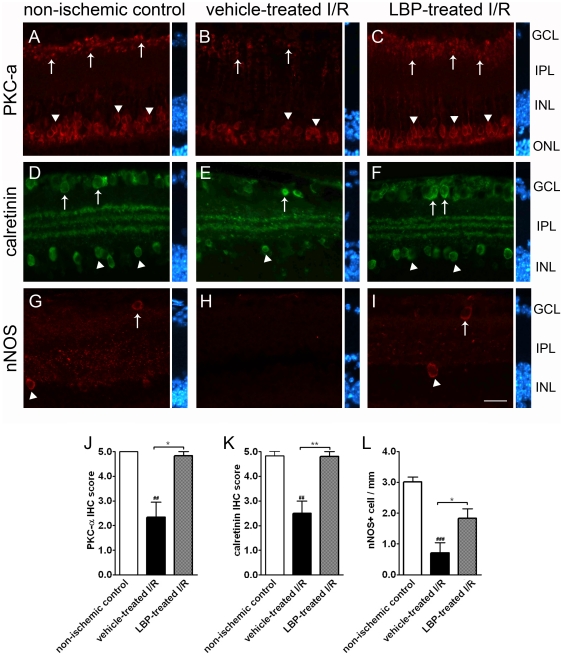
Immunoreactivity of cell markers for amacrine cells and bipolar cells in the LBP-treated retina was comparable with that in the non-ischemic control. (A–C) PKC-α Fewer cell bodies (arrow heads in B) and synaptic terminals (arrows in B) of rod bipolar cells were observed in the vehicle-treated I/R retina compared with those in the non-ischemic control retina (A) and LBP-treated I/R retina (C). Result is confirmed by semi-quantification of immunoreactivity of PKC-α (J). (D–F) Calretinin. Calretinin immunoreactivity was found in cell bodies of amacrine cells in GCL (arrows in D) and INL (arrow heads in D), and synaptic terminals which form three distinct strata in the IPL (D). I/R induced neuronal damage indicated by fewer calretinin-positive cell bodies (E) and disorganization of the strata in IPL of the vehicle-treated I/R retina (E). However, LBP pre-treatment prevented the damage (F). (K) Semi-quantification of calretinin immunoreactivity. (G–I) Neuronal nitric oxide synthase (nNOS). A small population of nNOS-positive amacrine cells was found in GCL (arrows in G & I) and INL (arrow heads G & I) of the non-ischemic control retina and LBP-treated I/R retina. However, these cells were scarcely seen in vehicle-treated I/R retina (H). (L) Quantification of nNOS-positive amacrine cells. ^##^p<0.01, ^###^p<0.001 vs. non-ischemic control retina; *p<0.05, **p<0.01 vs. vehicle-treated I/R retina. Scale bar, 25 µm. IHC score, immunohistochemistry score.

PKC-α is a marker for rod bipolar cells. Normally, the cell bodies of PKC-α-positive bipolar cells are located in the outer border of INL and their axons terminate at the inner border of IPL [27], [28]. The expression of PKC-α after retinal I/R was assessed semi-quantitatively using IHC score. The IHC scores in the vehicle-treated I/R retina, the LBP-treated I/R retina and non-ischemic control retina were 2.3±0.6, 4.8±0.2 and 5.0±0 arbitrary units, respectively (Fig. 3J). In other words, the expression of PKC-α in the vehicle-treated I/R retina was less when compared with that of the non-ischemic control retina (Fig. 3B). However, PKC-α expression in the LBP-treated I/R retina was more when compared with that in the vehicle-treated I/R retina (Fig. 3C).

### Amacrine cells

In non-ischemic control retina, calretinin-positive amacrine cells were present in GCL and the innermost layer of INL. The synaptic terminals of amacrine cells formed three distinct strata in IPL (Fig. 3D). In the vehicle-treated I/R retina, when compared with the non-ischemic control retina, the expression of calretinin appeared to be less in the two cellular layers; in addition, the innermost stratum in IPL appeared to be disorganized (Fig. 3E). In the LBP-treated I/R retina, when compared with the vehicle-treated I/R retina, the expression of calretinin appeared to be more; the three strata in IPL were present and distinct in the inner retina (Fig. 3F). The IHC scores in the vehicle-treated I/R retina, the LBP-treated I/R retina and the non-ischemic control retina were 2.5±0.5, 4.8±0.2 and 4.8±0.2 arbitrary units, respectively (Fig. 3K).

In the normal retina, a small population of amacrine cells expresses nNOS [28]. There were fewer nNOS-expressing amacrine cells found in the vehicle-treated retina as compared with the non-ischemic control retina (0.7±0.3 vs. 3.0±0.2 cells/mm, p<0.001) (Fig. 3H & 3L). There were more nNOS-expressing amacrine cells found in the LBP-treated I/R retina as compared with the vehicle-treated I/R retina (1.8±0.3 vs. 0.7±0.3 cells/mm, p<0.05) (Fig. 3I & 3L).

### Immunoreactivity of glial fibrillary acidic protein (GFAP)

**Figure 4:**
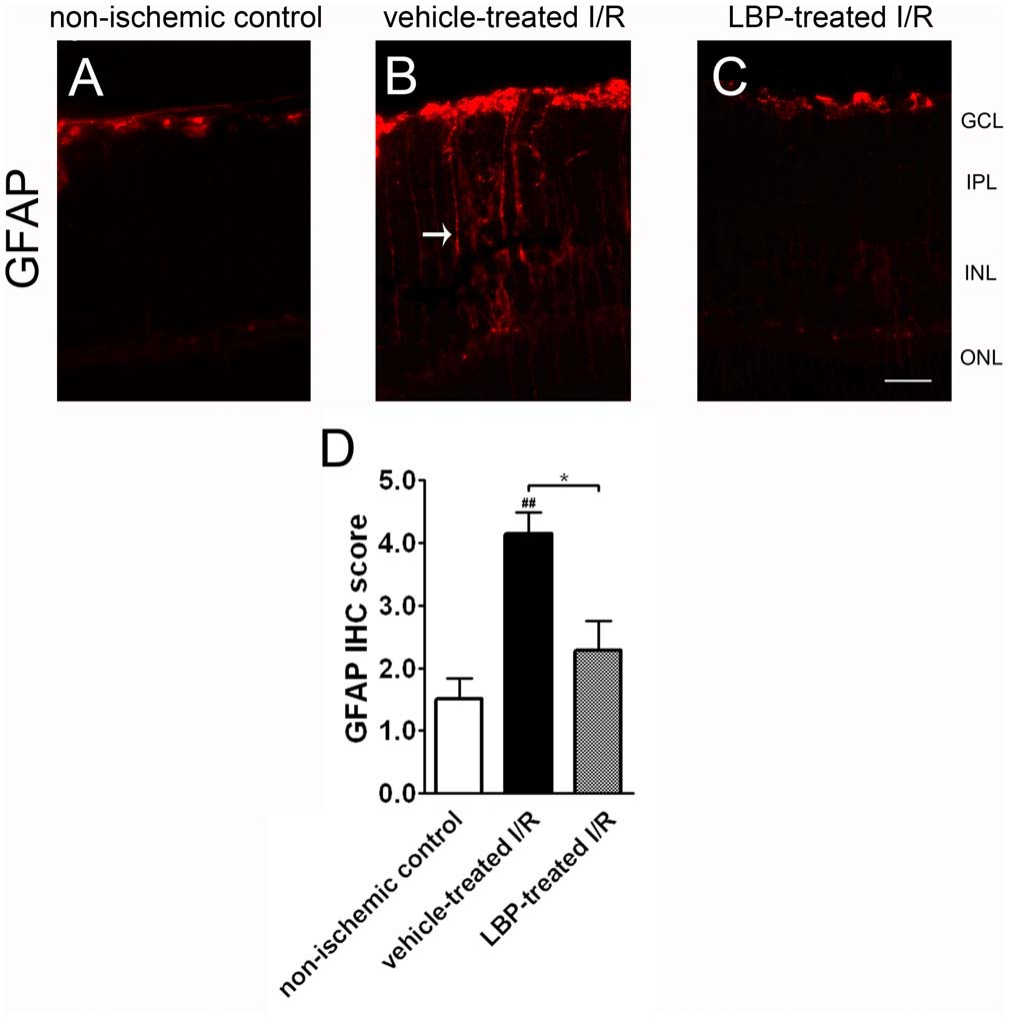
Lower level of glial fibrillary acidic protein (GFAP) activation in the LBP-treated I/R retina. (A–C) Immunohistochemistry of GFAP. I/R induced intense GFAP immunoreactivity in Muller cell processes of the vehicle-treated I/R retina (arrows in B), but not of the LBP-treated I/R retina (C). Results were confirmed by semi-quantification of IHC in (D). ^##^p<0.01 vs. non-ischemic control retina; *P<0.05 vs. vehicle-treated I/R retina. Scale bar, 25 µm.

In non-ischemic control retina, the expression of GFAP was confined to astrocytes in GCL (Fig. 4A). In the vehicle-treated I/R retina, GFAP immunoreactivity was markedly increased and was not just confined to the GCL but also found in the Muller cell processes (Fig. 4B). In the LBP-treated retina, in contrast to the vehicle-treated I/R retina, GFAP immunoreactivity was not increased (Fig. 4C). The IHC scores in the vehicle-treated I/R retina, the LBP-treated I/R retina and the non-ischemic control retina were 4.1±0.3, 2.3±0.5 and 1.5±0.3 arbitrary units, respectively (Fig. 4D).

### Immunoreactivity of AQP4

**Figure 5:**
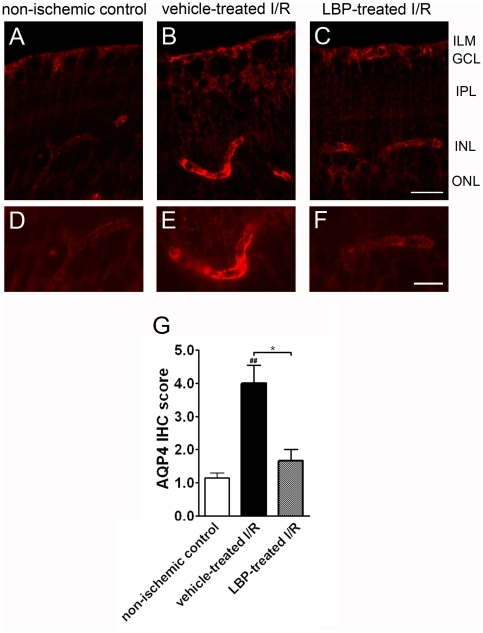
Lower level of aquaporin-4 (AQP4) immunoreactivity in the LBP-treated I/R retina. (A–C) Photomicrographs taken with a 40x objective. (D–F) Photomicrographs taken with a 60x objective. In the non-ischemic control retina, mild AQP4 immunoreactivity was observed in the astrocytic end-feet in ILM and perivascular regions near INL (A & D). However, intense AQP4 immunoreactivity was seen in the astrocytic end-feet in ILM and perivascular regions of the vehicle-treated I/R retina (B & E). However, the AQP4 immunoreactivity in LBP-treated I/R retina was comparable with that in the non-ischemic control retina (C & F). Results were further confirmed by the semi-quantitative analysis (G). ^##^p<0.01 vs. non-ischemic control retina, *p<0.05 vs. vehicle-treated I/R retina. Scale bar for A–C, 25 µm. Scale bar for D–F, 15 µm.

In the non-ischemic control retina, there was a weak immunoreactivity of AQP4 expressed in the astrocytic end-feet at the ILM and in the perivascular regions at the outer border of INL (Fig. 5A). The AQP4 staining was discontinuous along the blood vessels and appeared weak (Fig. 5A & 5D). In the vehicle-treated I/R retina, AQP4 immunoreactivity surrounding retinal vessels became more intense (Fig. 5B & 5E). In the LBP-treated I/R retina, in contrast showed no increased expression of AQP4 expression in the astrocytic end-feet and perivascular regions (Fig. 5C & 5F). The IHC scores in the vehicle-treated I/R retina, the LBP-treated I/R retina and the non-ischemic control retina were 4.0±0.5, 1.7±0.3 and 1.1±0.2 arbitrary units, respectively (Fig. 5G).

### Retinal blood vessel leakage

**Figure 6:**
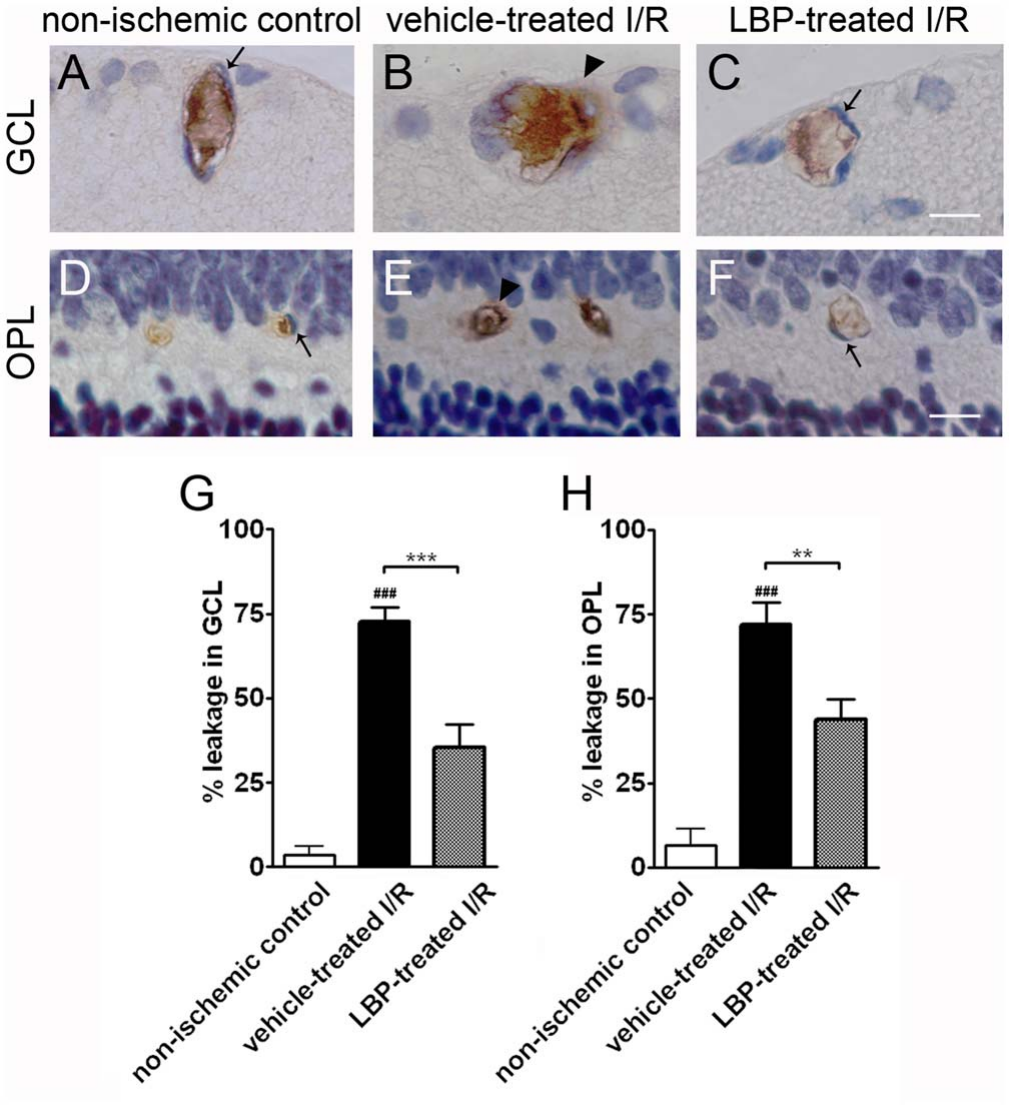
The number of leaky blood vessels in the LBP-treated I/R retina was comparable with that in the non-ischemic control retina. (A–C) IgG extravasations in blood vessels in GCL. (D–F) IgG extravasations in blood vessels in OPL. In non-ischemic control retina, IgG staining was confined inside the blood vessel lumen demarcated by endothelial cells (arrows in A & D). However, I/R induced blood vessel leakage in both GCL and OPL (arrow heads in B & E) in the vehicle-treated I/R retina but not in the LBP-treated I/R retina (arrows in C & F). (G, H) Quantification of blood vessel leakage in GCL (G) and OPL (H). More leaky blood vessels were observed in GCL (G) and OPL (H) of the vehicle-treated I/R retina. However, LBP treatment decreased the I/R-induced blood vessel damage, evident from fewer leaky blood vessels. ^###^p<0.001 vs. non-ischemic control retina; **p<0.01, ***p<0.001 vs. vehicle-treated I/R retina. Scale bar, 25 µm.

Blood vessel leakage was assessed by IgG extravasations. In the vehicle-treated I/R retina, when compared with the non-ischemic control, there were greater number of blood vessels with immuno-positive IgG signals located outside the endothelial vessel lining. These vessels were situated at the GCL (Fig. 6B & 6G) and OPL (Fig. 6E & 6H). In the LBP-treated I/R retina, when compared with the vehicle-treated I/R retina, there were smaller number of blood vessels showing leakage (Fig. 6C, 6F–H).

### Oxidative stress

**Figure 7:**
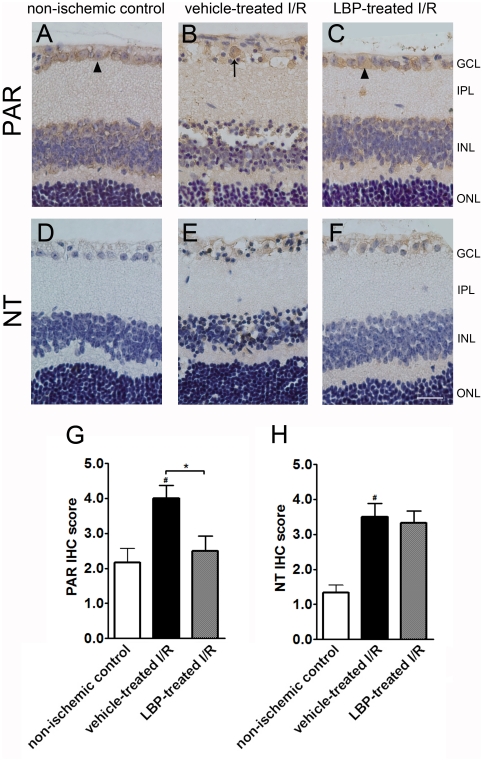
LBP pre-treatment lowered lipid peroxidation but not nitrosative stress in I/R retina. (A–C) Immunohistochemisty of poly(ADP-ribose) (PAR). PAR expression was localized in the cytosol of cells of the non-ischemic control retina and LBP-treated I/R retina (arrow heads in A & C). However, PAR expression was found inside the cell nuclei of the vehicle-treated retina (arrow in B). (D–F) Immunohistochemistry of nitrotyrosine (NT). A minimal NT expression was seen in the non-ischemic control retina (D). However, an increased level of NT immunoreactivity was noted in the vehcle-treated I/R retina and LBP-treated I/R retina (E & F). Results were confirmed by semi-quantification of IHC (G, PAR; H, NT). ^#^p<0.05 vs. non-ischemic control retina; *P<0.05 vs. vehicle-treated I/R retina. Scale bar, 25 µm.

Lipid peroxidation was evaluated using PAR immunohistochemistry. In non-ischemic control retina, PAR expression was limited inside the cytoplasm (Fig. 7A). A profound PAR immunoreactivity was found inside the cell nucleus in GCL which indicated an increased level of lipid peroxidation in the vehicle-treated I/R retina (Fig. 7B). However, LBP pre-treatment minimized the nucleus translocation of PAR expression (Fig. 7C). The IHC scores of PAR immunoreactivity in the vehicle-treated I/R retina, the LBP-treated I/R retina and the non-ischemic control retina were 4.0±0.4, 2.5±0.4 and 2.2±0.4 arbitrary units, respectively (Fig. 7G).

NT is a marker for nitrosative stress. Minimal NT immunoreactivity was noted in non-ischemic control retina (Fig. 7D). An increased level of NT expression was seen in the vehicle-treated I/R retina (Fig. 7E). However, LBP pre-treatment could not lower the NT expression in I/R retina (Fig. 7F). The IHC scores of NT immunoreactivity in the vehicle-treated I/R retina, the LBP-treated I/R retina and the non-ischemic control retina were 3.5±0.4, 3.3±0.3 and 1.3±0.2 arbitrary units, respectively (Fig. 7H).

## Discussion

BRB disruption, glial cell activation, oxidative stress and neuronal death are major concerns in retinal I/R injury. Neuroprotective agents that can retard or prevent these damages are beneficial in treating many ocular diseases in which retinal I/R is a complication. In our present study, we showed that pre-treating mice with LBP for 1 week could protect the animals from I/R injury by reducing neuronal cell death, retinal swelling, glial activation, BRB disruption and oxidative stress.

In the present study, unilateral retinal ischemia was induced by occluding the ICA which supplies blood to the ophthalmic artery [18], [19]. This model is one of the commonly used animal retinal I/R models [20], [21], [29]. Different from other retinal I/R model, the unilateral ICA occlusion model is a purely vascular model and does not involve any mechanical injury to the eye during the experimental operation [29]. The ophthalmic artery occlusion and blood resupply by ECA and ICA can be easily monitored by the duration of filament kept inside the vessel lumen. This model induces complete but reversible retinal ischemia injury [29] and leads to a considerable cell loss in inner retina by the blockade of ophthalmic artery [21]–[23], [30]. It mimics the clinical situation of transient monocular amaurosis fugax [21], [29] and other ocular diseases in which retinal I/R is a complication. Therefore, this artery occlusion model is a good tool for exploring neuroprotective agents against retinal I/R injury.

*Lycium barbarum* is a dried fruit that is used as a food or a medicine according to Chinese tradition [7]. It has been claimed that LBP can exhibit anti-aging, anti-tumor, cytoprotective, neuro-modulation, and immune modulation effects [7]. LBP can attenuate breakage of DNA by oxidation in the testicle cells in mice [31]. It has also been shown to protect DNA damage of peripheral blood lymphocytes against oxidative stress [32]. However, researches on the protective effects of LBP against ocular diseases are still ongoing. In the present study, we aim to look at the protective effects of LBP against retinal I/R injury. We focus on four aspects that are closely related to retinal I/R injury: anti-apoptosis, preservation of BRB integrity, prevention of retinal swelling, and anti-oxidation.

Retinal I/R induces neuronal death in the inner retina, especially RGC. An extensive loss of cells in the GCL has been seen after retinal I/R injury [21], [22]. A majority of these neurons die by apoptosis during retinal I/R injury [33]. Our data show an increased number of apoptotic nuclei in inner retinal layers, especially in the GCL, of the ischemic retina. The result is highly reproducible and comparable to our previously published data [22]. In previous studies, only a few TUNEL-positive apoptotic cells are found in inner retina with 1 hr ischemia injury although similar animal model is used [21], [30]. The discrepancy is probably due to the shorter ischemia period when compared with that in our study. Retinal function, assessed by the a-wave and b-wave of electroretinography, is deteriorated in the ischemic eye [29], suggesting that the function of retinal neurons is weakened in retinal I/R injury. Similar to a previous study [34], we also show that retinal I/R caused detrimental injury to amacrine cells, as indicated by the great reduction in calretinin expression and the dis-organization of IPL stratification. Apart from calretinin-expressing amacrine cells, fewer nNOS-expressing amacrine cells were observed in I/R retina [22], [35], [36]. Comparable phenomenon was noted in bipolar cell immunoreactivity in the present study. Apart from neuronal damage, I/R injury also induces impairment in synaptic connections of retinal neurons [30]. Vesl-1L/Homer 1c (V-1L) is a marker for used as a marker for the assessment of changes in synaptic connectivity preceding apoptosis and during early stages of apoptosis of RGCs [30]. A reduction of V-1L immunoreactivity indicates that the synaptic connections of RGCs and other inner retinal neurons, including amacrine cells and bipolar cells, are attenuated after I/R. In this study, our first aim to investigate the anti-apoptotic and neuroprotective effects of LBP in retinal I/R injury. Significantly fewer apoptotic cells were seen in GCL and INL of LBP-treated ischemic retina. The detrimental effects on amacrine cells and bipolar cells due to I/R injury was minimized. In outer retina, LBP has been shown to decrease apoptosis in photoreceptors of rd1 mice with photoreceptor degeneration [37]. Its beneficial protection is as good as other neuroprotective agents such as lutein and zeaxanthin [22], [37], [38], [39], [40]. Our data elucidated that LBP is anti-apoptotic not only in outer retina, but also inner retina. Moreover, our earlier studies showed that LBP protects against beta-amyloid peptide neurotoxicity by inhibiting pro-apoptotic signaling pathways such as JNK, PKR and caspase-3 [8], [12]. It is therefore likely that LBP may exert its neuroprotective effects via inhibition of JNK, PKR and caspase-3 activity in retinal I/R injury.

BRB is a protective barrier which consists of the outer and inner BRB. The role of BRB is to maintain the homeostatic condition of retinal microenvironment and exclude harmful substance getting into the retina [41], [42]. The outer barrier is formed by the retinal pigment epithelium, separating the outer retina from the choroid [43]. The inner BRB is formed by the tight junctions of the vascular endothelial cells and sheathed by the Muller cell processes [43], [44]. In many ocular disease including ischemic retinal vein/artery occlusion and diabetic retinopathy, breakdown of the inner BRB increases retinal vascular permeability, resulting in retinal edema and cell death [26], [42], [45]. In the present study, we examined BRB integrity by assessing blood vessel leakage using IgG immunohistochemistry. The majority of IgG was confined in the lumen of blood vessels in normal retina. However, IgG extravasations were present outside the vessel lumen after I/R injury, indicating blood vessel leakage and BRB breakdown. IgG extravasations was minimized in ischemic retina treated with LBP. This suggests that LBP could prevent or minimize the disruption of BRB in retinal I/R injury, which has not been studied previously.

Disruption of BRB leads to swelling of astrocytes and Muller cells processes associated with the activation of GFAP and AQP4 under ischemic conditions [41], [42]. These causes further retinal edema and tissue damage. In normal conditions, water and ion homeostasis in retina is controlled by Muller cells via the transmembrane aquaporin water channels [6], [46]. Muller cells do not express GFAP under normal physiological situations. The end feet of retinal astrocytes and Muller cells wrap around the blood vessels of the superficial retina and express AQP4 [2], [6], [46]. GFAP up-regulation is a hallmark of astrocyte and Muller cell activation and the resulting reactive gliosis [47]. Expression of GFAP in Muller cells and astrocytes occurs under pathological conditions such as I/R injury [48], [49]. In addition, over-expression of AQP4 is associated with water influx into the retina during injury [1]. Water influx is further exaggerated during reperfusion. Swelling and hypertrophy of Muller cells occur consequently upon overloading of intracellular K^+^ and the movement of water inside cells [1], [50]. It has been shown that the deletion of AQP4 gene can protect retina against swelling in a mouse model of ischemia, thus emphasizing the importance of AQP4 in water transport [51]. In the present study, activation of GFAP and AQP4 was observed in the vehicle-treated retina, indicating the role of Muller cells and AQP4 controlling water transport in retina. Moreover, retinal swelling was noted in ischemic retina. These results indicate a close association among the BRB integrity, the control of water flux and retinal swelling. Here, we show that LBP pre-treatment could diminish the activation of GFAP and AQP4, as well as retinal swelling in retinal I/R injury, implying the inhibitory effects of LBP in retinal swelling by minimizing the activation of Muller cell and AQP4.

Oxidative stress plays a role in retinal I/R injury due to the high content of polyunsaturated fatty acid in retina. Reactive oxygen species (ROS) and free radical formation during I/R facilitates lipid peroxidation of membrane, denature of protein and DNA damage [52], [53]. The breakdown of DNA strands activates the nuclear enzyme poly(ADP-ribose) polymerase (PARP) to produce PAR [54]. PARP cleaves nicotinamide adenine dinucleotide, and subsequently leads to energy failure and cell death [55], [56]. Another pathway of inducing oxidative stress is the nitrosative injury. Free radical formation facilitates nitric oxide (NO) production, which reacts with superoxide to from peroxynitrite, a strong oxidant [57]. Peroxynitrite leads to nitration of tyrosine residues of cells to form NT [58], [59]. Therefore, NT is an indicator for oxidative-nitrosative stress. Increased immunoreactivity of PAR and NT was observed in the vehicle-treated I/R retina, indicating increased oxidative stress associated with retinal I/R. In addition, oxidative stress has been shown to have an injurious role in BRB integrity by disruption of tight junctions in retina [60]. ROS generated in ischemia up-regulates vascular endothelial growth factor (VEGF) gene expression [61]. Tight junction damage and increased VEGF expression attribute to an increase in vascular permeability and subsequent BRB breakdown [41]. Treatment with anti-oxidants, including *Lycium barbarum*, increases glutathione peroxidase activity and glutathione levels, and decreases cystine concentrations in retina of *rd1/rd1* mice, indicating a decreased level of oxidative stress in retinal generation [37]. In the present data, LBP-treated I/R retina had a less extent of oxidative stress, indicated by a decreased level of PAR immunoreactivity. These findings suggest that one of the possible mechanisms for LBP to exhibit its cytoprotective effects is via attenuation of oxidative stress. The decrease in oxidative stress may attribute to prevent or diminish BRB breakdown induced by I/R injury. However, LBP treatment did not lower NT immunoreactivity after I/R injury. This may imply that LBP does not have anti-oxidative effect against nitrosative stress. Further investigation is required to confirm this observation.

Increasing lines of evidence have demonstrated that modulation of immune responses can affect the degenerative processes of neurons in the CNS [62], [63]. In fact, previous studies have shown that LBP can enhance immune function [9], [10], [64], suggesting that the beneficial effects of LBP may also be mediated by immuno-modulations. Our recent study also demonstrated that LBP protect RGC by modulating the activation of microglia in an animal model of chronic ocular hypertension [65]. We speculate that neuroprotection conferred by LBP may be a combination of many different mechanisms including anti-apoptosis, anti-oxidation and immuno-modulation in addition to our current findings i.e. protection of the BRB and maintenance of the water homeostasis via the down-regulation of AQP4.

### Conclusions

Our data indicated that pre-treatment with LBP for 1 week could effectively protect the mouse from retinal I/R injury. The present study hence suggests that LBP may be used as a preventive TCM for diseases associated with I/R such as amaurosis fugax and acute glaucoma. Further studies are warranted to evaluate the therapeutic effects of LBP and other potential neuroprotective mechanisms.
